# Meningitis due to *Roseomonas* in an immunocompetent adolescent

**DOI:** 10.1099/acmi.0.000213

**Published:** 2021-03-10

**Authors:** Rabbia S. Waris, Melissa Ballard, David Hong, Talal B. Seddik

**Affiliations:** ^1^​ Pediatric Hospital Medicine, Stanford University, Stanford, CA, USA; ^2^​ Pediatric Neurosurgery, Lucile Packard Children’s Hospital, Stanford, CA, USA; ^3^​ Pediatric Neurosurgery, Stanford University, Stanford, CA, USA; ^4^​ Pediatric Infectious Diseases, Stanford University, Stanford, CA, USA

**Keywords:** 16S rRNA sequencing, Aseptic meningitis, Chemical meningitis, Meningitis, Neurosurgery, Roseomonas

## Abstract

Both bacterial and aseptic meningitis can complicate neurosurgery, but they are often difficult to distinguish clinically or by cerebrospinal fluid (CSF) analysis. We present an adolescent with subacute meningitis after neurosurgery, eventually diagnosed with meningitis caused by *Roseomonas mucosa* via 16S rRNA gene sequencing after two negative CSF cultures. He was treated successfully with intravenous meropenem with full recovery. This case shows that distinguishing bacterial from aseptic meningitis is important to allow directed antibiotic therapy. We recommend considering bacterial meningitis in the differential diagnosis of aseptic meningitis complicating neurosurgery, and to perform molecular diagnostics such as bacterial sequencing if the suspicion of bacterial meningitis is high.

## Introduction

Meningitis is a known complication of neurosurgical procedures. Aseptic meningitis (also known as chemical meningitis) after neurosurgical procedures is diagnosed when clinical presentation and cerebrospinal fluid (CSF) analysis results are consistent with meningitis, but bacterial cultures are negative [1]. It is often difficult to differentiate between aseptic and bacterial meningitis based on clinical presentation and CSF chemistry and cytology [1]. Molecular microbiologic techniques, such as bacterial 16S rRNA PCR amplification and sequencing, are now increasingly utilized when infection is highly suspected and cultures are non-diagnostic. Hence, they could be used in cases where the suspicion for bacterial meningitis is high, yet bacterial cultures from the CSF are negative [2].

*Roseomonas* species is a Gram-negative, slow growing, pink-pigmented, coccoid bacteria that was first described in 1993 [3]. Infections due to *Roseomonas* sp. are uncommon, and most species are isolated from environmental samples [4]. Most clinically significant infections have been described in patients with central venous catheters, immune-compromised status or underlying chronic conditions. Certain *Roseomonas* clades are human microbiota and pathogens, mainly isolated from skin and respiratory tract, which explains their tendency to cause central-line associated bloodstream, skin and soft tissue infections [4]. The main pathogenic species include *Roseomonas mucosa, Roseomonas gilardii* and *Roseomonas cervicalis *. Central nervous system (CNS) infection caused by *Roseomonas* sp. is rarely reported in the literature; in fact, *Roseomonas* sp. were considered contaminants in one study of neonatal meningitis [5]. Examples of reported CNS infections due to *Roseomonas* include: ventriculitis in a 54-year-old male after ventriculostomy and aneurysm clipping for subarachnoid hemorrhage [6], and neonatal meningitis without any details on presentation [7].

Herein, we present a case that highlights the difficulty in distinguishing between aseptic and bacterial meningitis after neurosurgery and diagnosing *Roseomonas* sp. infections.

## Case report

A 17-year-old male presented to our emergency department (ED) with severe headache, nausea, emesis and fever for 1 day. Medical history was notable for chronic headache for 1 year eventually attributed to a large frontotemporal arachnoid cyst with mass effect. He underwent left temporal craniotomy for open fenestration of the arachnoid cyst 25 days prior to this presentation. He had a mild headache postoperatively, attributed to aseptic meningitis, that improved with steroids. No drains were left postoperatively. He was discharged on postoperative day (POD) 3 with steroid taper for 10 days. Overall, he continued to have intermittent headache that were not severe enough to seek medical attention until this presentation.

In ED, his work-up revealed positive influenza B PCR, unremarkable CBC, C-reactive protein 51 mg L^−1^ (normal≤9.9 mg L^−1^) and erythrocyte sedimentation rate 25 mm h^−1^ (normal ≤15 mm h^−1^). Head CT scan showed stable postoperative findings. He was admitted for supportive care but noted to have progressively worsening left temporal swelling at the craniotomy site that was diagnosed as a CSF leak from displaced dural graft secondary to persistent emesis. On the third day of admission, he underwent repeat craniotomy, revealing a defect in the dura that was repaired with a new graft and dural sealant. Although there were no findings suggestive of infection, a surveillance wound culture was obtained from soft tissue surrounding the craniotomy flap. Postoperatively, his symptoms improved except for mild headaches and he was discharged on POD 1 after a stable brain MRI.

The soft tissue swab was inoculated to 5 % sheep blood agar (BBL catalogue no. 221261), Chocolate agar (BBL catalogue no. 221267), and MacConkey agar (BBL catalogue no. 221270). The Gram stain prepared from the tissue swab showed no polymorphonuclear cells or organisms on the slide. The incubation conditions for the primary culture plates and subcultures were 5 % CO_2_ atmosphere at 35°C. Light growth of pinpoint Gram-negative coccoid colonies appeared at 48 hours, with poor growth at 72 hours. After 96 hours incubation, pale salmon-coloured colonies grew on 5 % sheep blood agar and chocolate agar. These were tested for identification and susceptibilities using the NC68 panel (Beckman Coulter catalogue no. B1017-422) on the MicroScan WalkAway 96 Plus System (Beckman Coulter), but there was insufficient growth on the panel for identification or susceptibility results due to the organism’s fastidious, slow-growing properties on culture. A Gram-negative identification card (bioMérieux Vitek 2 GN card, catalogue no. 21341) was set up on the VITEK 2 system (bioMérieux) and *Roseomonas gilardii* was identified with 98 % confidence. Table 1 shows the minimum inhibitory concentration (MIC) and susceptibility for the tested antibiotics. Susceptibility testing was done utilizing Etest (bioMérieux) strips. Susceptibility results were interpreted according to the Clinical and Laboratory Standards Institute [8]; the organism was susceptible to both levofloxacin and meropenem.

**Table 1.** Antibiotic susceptibility testing and interpretation of the *Roseomonas* sp. isolate from soft tissue swab

Antibiotic	MIC	Interpretation
Meropenem	0.12 µg ml^−1^	Susceptible
Levofloxacin	0.12 µg ml^−1^	Susceptible

The patient was called back to the ED on POD 5 when the organism was identified on soft tissue culture; susceptibility testing was pending at that point. He reported mild photophobia and intermittent headache since the dural repair. His exam did not show signs of meningismus or nuchal rigidity. CSF studies revealed WBC 231/mm^3^ (normal 0–5/mm^3^), 51 % neutrophils, 47 % lymphocytes, 2 % monohistiocytes, RBC 16/mm^3^ (normal 0/mm^3^), protein 77 mg dl^−1^ (normal 15–45 mg dl^−1^), glucose 41 mg dl^−1^ (normal 40–70 mg dl^−1^) and a negative Gram stain. Given the abnormal CSF profile, differential diagnosis included bacterial and aseptic meningitis. In the case of aseptic meningitis, the growth of *Roseomonas gilardii* from the soft tissue sample would be attributed to specimen contamination or soft tissue infection. He received intravenous (IV) meropenem 2 grams every 8 hours for possible bacterial meningitis with *Roseomonas* sp. and dexamethasone for possible aseptic meningitis while CSF cultures were pending. He showed rapid improvement. After his blood and CSF cultures were negative for 48 hours; he was diagnosed with aseptic meningitis and soft tissue infection with *Roseomonas gilardii *. He was discharged on oral levofloxacin 750 mg daily for 6 days and a steroid taper for 7 days. CSF bacterial cultures and 16s rRNA PCR and sequencing, for suspicion of *Roseomonas* sp. infection as described below, failed to identify a pathogen. The CSF sample volume for culture was more than 1 ml, the incubation conditions were 5–10 % CO_2_ atmosphere at 35°C and the culture was held for 10 days.

Two days after completion of antibiotics and steroid course (POD 15), he presented to the ED again with severe headache, nausea, emesis, neck pain, photophobia and fever for 1 day. He had nuchal rigidity without other signs of meningismus. Repeat CSF analysis showed WBC 8720/mm^3^, 76 % neutrophils, 4 % lymphocytes, 20 % monohistiocytes, RBC 8/mm^2^, protein 213 mg dl^−1^, glucose 22 mg dl^−1^. Complete blood count was notable for WBC 30×10^3^ µl^−1^ with 77 % neutrophils. Brain MRI and MRV with contrast showed generalized leptomeningeal enhancement including ventricles. He was re-admitted and started on IV meropenem 2 grams every 8 hours and empiric vancomycin 15 mg/kg/dose every 8 hours. His repeat CSF culture was negative. Again, the CSF specimen was >1 ml in volume with the same incubation conditions and duration as described above. Vancomycin was discontinued after 48 hours of negative CSF culture, while meropenem was continued pending further testing given the higher suspicion for *Roseomonas* sp. infection. CSF bacterial 16s rRNA PCR and sequencing identified *Roseomonas* sp. consistent with bacterial meningitis. In both instances, the previous and current CSF testing, 16S rRNA amplicon sequencing was performed with universal primers (5F TTGRAGAGTTTGATYMTGGCT and 531R GTATTACCGCGGCKGCTG) using cycle sequencing described before [9]. Percent identity was 99.8 % (339/440) to *Roseomonas mucosa* strain B_MAR_18_176 (GenBank: MN396262.1)

The patient’s symptoms resolved within a week of antibiotic therapy, and he was discharged with meropenem 2 g IV every 8 hours and oral dexamethasone taper for 4 weeks. He was followed weekly in the outpatient clinic with complete resolution of all his symptoms including headache.

## Discussion

Our patient presented with subacute headache and had multiple surgeries and lumbar punctures. Given that he had headache throughout the presentation, it is difficult to ascertain the timing of infection, and whether it was introduced during initial surgery, afterwards through the CSF leak, or during CSF leak repair. Although two CSF cultures were negative, identification of the same rare pathogen (* Roseomonas* sp.) from soft tissue swab from craniotomy flap by culture and, less than 2 weeks later, from CSF by 16S rRNA sequencing is consistent with bacterial meningitis with this opportunistic pathogen. In addition, the clinical response to antibiotics targeted against *Roseomonas* sp. provides further evidence of causation. We note that the species of *Roseomonas* was different between the first specimen obtained from soft tissue swab culture and identified by VITEK 2 system (* Roseomonas gilardii *) and the second specimen obtained from the CSF via 16S rRNA amplicon sequencing (* Roseomonas mucosa *). However, this difference between phenotypic and molecular identification is well documented and not uncommon [10]. Given that the VITEK 2 system utilizes phenotypic identification, it is much more likely that the 16S rRNA amplicon sequencing identification was more accurate and that both isolates were *Roseomonas mucosa* [10]. Unfortunately, the first isolate from soft tissue swab identified as *Roseomonas gilardii* by VITEK 2 system was not available for molecular comparison with the second isolate identified from the CSF as *Roseomonas mucosa* by sequencing. However, it is highly unlikely that there were two different species isolated from adjacent sites given the rarity of this organism in clinical samples.

Bacterial meningitis is a rare complication of neurosurgery, complicating 0.3–1.9 % of cranial surgeries [11]. Aseptic meningitis after neurosurgery is a separate entity diagnosed when CSF cultures are negative. Although it was first described in the early 1920s by Cushing *et al*., little is known about its pathogenesis. Proposed pathogenesis, including inflammation caused by breakdown of RBCs or from surgical materials, is nebulous and unproven [1]. Further, both aseptic and bacterial meningitis after neurosurgery present with fever, meningismus, headache and CSF pleocytosis, making them difficult to differentiate clinically or by CSF chemistry and cytology [1]. Treatment is empiric and symptomatic as the condition remains a diagnosis of exclusion in the setting of negative cultures. However, it is not uncommon for patients with negative CSF cultures to receive antibiotics due to clinical concern for bacterial meningitis or while cultures are pending [1].

Molecular microbiologic techniques, such as bacterial 16S rRNA PCR amplification and sequencing, are now increasingly utilized when infection is suspected and cultures are negative. Cultures can be falsely negative due to antimicrobial therapy prior to specimen collection, low pathogen load in the sample or infection with organisms that are difficult to grow in conventional culture media. To investigate the involvement of bacteria in the pathogenesis of aseptic meningitis after neurosurgery, Druel *et al*. performed bacterial 16S rRNA PCR amplification on CSF from patients with aseptic meningitis after craniotomy [2]. Interestingly, all patients diagnosed with ‘aseptic meningitis’ after craniotomy had detected bacterial RNA in CSF, while controls without evidence of meningitis after craniotomy had no evidence of bacterial RNA in CSF. Furthermore, cycle threshold for bacterial RNA detection from ‘aseptic meningitis’ cases was higher than positive controls with bacterial growth by CSF culture, denoting lower bacterial load in ‘aseptic meningitis’ cases. This implies that what was thought to be aseptic meningitis after neurosurgery, is caused by bacteria in low concentration in CSF, preventing detection by CSF cultures [2].

*Roseomonas* sp. is a qualified candidate to cause infections with negative bacterial culture, given its fastidious nature and slow growth [12]. This is observed in our patient, with growth from soft tissue after more than 48 hours of incubation, and no growth from two CSF cultures held for 10 days. Fanella *et al*. reported a similar case of a 16-year-old girl with subacute postoperative septic arthritis of the knee, in which *Roseomonas* sp. was identified only on the third culture of the synovial fluid and final diagnosis of *Roseomonas gilardii* was made via 16S rRNA gene sequencing [13]. This case and our case highlight the high degree of suspicion needed to diagnose *Roseomonas* infection in patients with postsurgical subacute manifestations. Further, it is especially important to identify *Roseomonas* as a pathogen given its resistance to cephalosporin and penicillin, the main antibiotics used empirically [14]. Our *Roseomonas* isolate from swab culture was susceptible *in vitro* by E-test to levofloxacin and meropenem, and the patient responded clinically to oral levofloxacin and meropenem.

Although *Roseomonas* is a rare CNS pathogen, it is important to consider it in cases of subacute meningitis complicating neurosurgical interventions with negative CSF cultures. We recommend early consideration of PCR testing in postoperative cases of meningitis to avoid prolonged complication management and additional neurologic sequelae.
